# Comparative Proteomic Analysis Provides New Insights Into Low Nitrogen-Promoted Primary Root Growth in Hexaploid Wheat

**DOI:** 10.3389/fpls.2019.00151

**Published:** 2019-02-20

**Authors:** Yanhua Xu, Yongzhe Ren, Jingjing Li, Le Li, Shulin Chen, Zhiqiang Wang, Zeyu Xin, Feng Chen, Tongbao Lin, Dangqun Cui, Yiping Tong

**Affiliations:** ^1^State Key Laboratory of Wheat and Maize Crop Science, Collaborative Innovation Center of Henan Grain Crops, College of Agronomy, Henan Agricultural University, Zhengzhou, China; ^2^Department of Life Sciences, Shangqiu Normal University, Shangqiu, China; ^3^State Key Laboratory of Plant Cell and Chromosome Engineering, Institute of Genetics and Developmental Sciences, Chinese Academy of Sciences, Beijing, China

**Keywords:** *Triticum aestivum* L., primary root, low nitrogen, proteome, iTRAQ

## Abstract

Nitrogen deficient environments can promote wheat primary root growth (PRG) that allows for nitrogen uptake in deep soil. However, the mechanisms of low nitrogen-promoted root growth remain largely unknown. Here, an integrated comparative proteome study using iTRAQ analysis on the roots of two wheat varieties and their descendants with contrasting response to low nitrogen (LN) stress was performed under control (CK) and LN conditions. In total, 84 differentially abundant proteins (DAPs) specifically involved in the process of LN-promoted PRG were identified and 11 pathways were significantly enriched. The Glutathione metabolism, endocytosis, lipid metabolism, and phenylpropanoid biosynthesis pathways may play crucial roles in the regulation of LN-promoted PRG. We also identified 59 DAPs involved in the common response to LN stress in different genetic backgrounds. The common responsive DAPs to LN stress were mainly involved in nitrogen uptake, transportation and remobilization, and LN stress tolerance. Taken together, our results provide new insights into the metabolic and molecular changes taking place in contrasting varieties under LN conditions, which provide useful information for the genetic improvement of root traits and nitrogen use efficiency in wheat.

## Introduction

Nutrient deficiencies are major limiting factors for crop yield worldwide. The root system is the main organ of plant nutrient uptake from soil, and improvements in root traits herald a new green revolution and further yield increases (de Dorlodot et al., 2007; Lynch, 2007; Den Herder et al., 2010). Plant roots have high plasticity under different environmental conditions (Linkohr et al., 2002; Hochholdinger and Tuberosa, 2009; Gruber et al., 2013; Lynch et al., 2014; Schmidt and Gaudin, 2017). In environments where nitrogen is limiting, the growth of primary and lateral roots is promoted, enabling them to reach the deeper soil layers. As nitrate is highly mobile and easily leached, the deeper soil layers may hold plentiful nitrogen supplies (Linkohr et al., 2002; Gruber et al., 2013; Lynch, 2013). Therefore, the ability to develop a deep root system under conditions of low nitrate availability is of vital importance for nitrogen acquisition. Since primary roots determine the depth of the root system to a great extent, especially at wheat seedling stage, it is important to study the response of primary roots to low nitrogen (LN) stress. It has been shown that the response of roots to LN stress is significantly different among different genotypes (Saengwilai et al., 2014; Ren et al., 2017). The acquisition of plants with different genetic backgrounds and contrasting phenotypes to LN stress laid the foundation for further research on the molecular mechanisms of LN-promoted PRG.

In *Arabidopsis*, the auxin biosynthesis pathway and some small peptides, present as signal molecules, are involved in the response of plant roots to LN levels (Bisseling and Scheres, 2014; Ma et al., 2014). Recent studies have shown that LN-induced rice primary root elongation is regulated by nitric oxide (NO) and strigolactones (SLs) signaling pathways (Sun et al., 2016; Sun H. W. et al., 2017). In wheat, overexpression of the *TaNFYA-B1* gene, which encodes one of the subunits of transcription factor NF-Ys, significantly increases total root length, root biomass, nitrogen uptake, and grain yield of transgenic plants (Qu et al., 2015). Also, overexpression of *TaNAC2-5A* in wheat enhances root growth and nitrate influx rate, and hence increases the roots' ability to acquire nitrogen (He et al., 2015). However, despite the progress that has been made, the molecular mechanisms involved in LN-promoted PRG in wheat remain largely unknown.

We previously developed a recombinant inbred lines (RIL) population derived from two elite winter wheat varieties, XY54 and J411. XY54 has been shown to have a larger and deeper root system than J411 (Ren et al., 2012). Further study revealed that the response of the primary roots of XY54 and J411 to LN stress exhibited contrasting phenotypes. The maximum root length (MRL) of XY54 under LN conditions was significantly longer than that under normal nitrogen supply conditions (CK), but not in J411. Furthermore, there existed significant segregation among the RILs (Ren et al., 2017). Here, we selected 15 lines exhibiting significantly enhanced primary root growth (PRG) under LN conditions and another 15 lines exhibiting no obvious induction of PRG by LN stress from the RIL population. The groups were termed the LRG (*L*onger *R*oot *G*rowth of primary root promoted by LN stress) group and the SRG (Shorter Root Growth of primary root promoted by LN stress) group, respectively. We performed an integrated comparative proteome study using iTRAQ analysis on the roots of the SRG and LRG groups, and their parents (XY54 and J411) under control (CK) and low nitrogen (LN) conditions. This work identified 84 DAPs specifically involved in the process of LN-promoted PRG and 59 DAPs involved in the common response to LN stress in different genetic backgrounds. Significantly enriched pathways were identified and discussed.

## Materials and Methods

### Plant Materials

XY54 and J411 are two Chinese winter wheat varieties with contrasting phenotypes to LN stress. There is no significant difference between the aboveground biomass of XY54 and J411, but the nitrogen uptake of XY54 is significantly higher than that of J411 (Zhang et al., 2005). We had previously developed a RIL population derived from the cross of XY54 and J411. To minimize differences in genetic background, we selected 15 lines exhibiting enhanced PRG under LN conditions (the LRG group) and another 15 lines exhibiting no obvious induction of PRG by LN stress (the SRG group) from the RIL population, together with their parents XY54 and J411, as materials for comparative proteomic analysis to identify candidate proteins and pathways involved in the regulatory network of the LN-promoted PRG process.

### Plant Growth Conditions and Evaluation of Root Morphology

Two independent trials were conducted for further phenotypic analysis (Trial 1) and comparative proteomics studies (Trial 2), respectively. In both trials, plants were randomly placed and grown in a greenhouse. Seed sterilization, germination, and the growth conditions of wheat plants were conducted according to Ren et al. (2012). Ca(NO_3_)_2_ was used as the only nitrogen source. The nutrient solutions were prepared according to Li et al. (2011). Appropriate amount of CaCl_2_ was added to each low N nutrient solution to balance the calcium concentration in the normal and low N nutrient solutions (Li et al., 2011).

In Trial 1, germinated XY54 and J411 seeds with residual endosperm removed were transferred to eight 13-liter-plastic pots, each containing nutrient solution with different nitrogen supply levels (0, 0.05, 0.1, 0.2, 0.5, 1.0, 2.0, and 4.0 mM ${\text{NO}}_{3}^{-}$, respectively). The nutrient solution was refreshed every 2 days and its pH was adjusted to 6.0 before refreshing. The MRL of XY54 and J411 were measured using a ruler at 25 d after transfer with at least four replications in all treatments. The developmental stages of wheat plants ranged from Zadoks growth scale 12–22 under different nitrogen supply levels (Zadoks et al., 1974).

In Trial 2, germinated seeds of lines in the LRG group, SRG group, and their parents XY54 and J411 (with residual endosperm removed) were transferred to CK (2.0 mM ${\text{NO}}_{3}^{-}$) and LN (0.1 mM ${\text{NO}}_{3}^{-}$) nutrient solutions. The MRL of each plant was measured at 12 d after transfer. The developmental stages of the wheat plants were Zadoks growth scale 12 and 11 under CK and LN conditions, respectively (Zadoks et al., 1974). The whole seminal roots of 15 individual plants from the LRG group, SRG group, XY54 and J411 were harvested and pooled, respectively. The root samples were frozen with liquid nitrogen and stored at −80°C for protein and RNA extraction. This collection was repeated three times as biological replicates.

### Protein Extraction, Digestion, and iTRAQ Labeling

**Figure 1:**
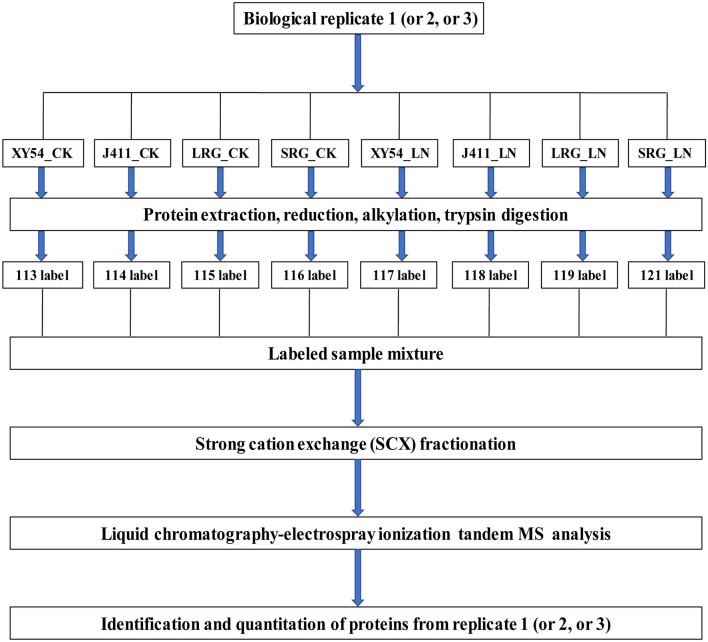
iTRAQ 8-plex labeling and LC-MS/MS workflow for identifying proteins in wheat roots under control (CK) and Low nitrogen (LN) conditions. Three biological replicates were performed. For each biological replication, one eight-plex iTRAQ set was used for the eight samples. The root samples of XY54, J411, longer root growth promoted by low nitrogen (LRG) group, and shorter root growth promoted by low nitrogen (SRG) group under control (CK) and low nitrogen (LN) conditions were named as XY54_CK, J411_CK, LRG_CK, SRG_CK, XY54_LN, J411_LN, LRG_LN, and SRG_LN, respectively.

Protein extraction was conducted according to a previously reported protocol (Thiellement et al., 2010; Hu et al., 2015; Zhao et al., 2016). Protein digestion was conducted using the FASP procedure (Wisniewski et al., 2009; Hu et al., 2015; Zhao et al., 2016). In brief, each protein sample was digested in a 40 μL trypsin solution overnight at 37°C. The ratio of trypsin to protein is 1:50 (w/w). The products of the digestion were collected as a filtrate and desalted on C18 Cartridges (Empore™ SPE Cartridges C18, Sigma), concentrated by vacuum centrifugation and dissolved in 40 μl of 0.1% (v/v) formic acid. The peptide content was determined by UV light spectral density at 280 nm using an extinctions coefficient of 1.1 of 0.1% (g/l) solution. Finally, 100 μg peptide mixture of each sample was labeled with iTRAQ reagents according to the manufacturer's instructions (Applied Biosystems, USA). As described in Figure 1, XY54_CK, J411_CK, LRG_CK, SRG_CK, XY54_LN, J411_LN, LRG_LN, and SRG_LN were labeled with 113, 114, 115, 116, 117, 118, 119, and 121, respectively. For each biological replicate, one eight-plex iTRAQ set was used for the eight samples (Figure 1). Three biological replicates were conducted to minimize experimental errors.

### Strong Cation Exchange (SCX) Fractionation

iTRAQ labeled peptides were combined and dried under vacuum. Strong cationic exchange (SCX) chromatography was performed to fractionate the labeled peptides using the AKTA Purifier system (GE Healthcare) as previously described (Hu et al., 2015; Zhao et al., 2016) with minor modifications. In brief, the labeled peptide mixture was acidified using buffer A (10 mM KH_2_PO_4_ in 25% of ACN, pH 3.0) and eluted at a flow rate of 1 ml/min with a gradient of 0–8% buffer B (500 mM KCl, 10 mM KH_2_PO_4_ in 25% of ACN, pH 3.0) for 22 min, 8–52% buffer B during 22–47 min, 52–100% buffer B during 47–50 min, 100% buffer B during 50–58 min, and buffer B was reset to 0% after 58 min. The elution was monitored by absorbance at 214 nm, and fractions were collected every 1 min. Thirty fractions were collected and merged into 10 parts (fraction 1, fraction 2, fraction 3 were combined to part 1; fraction 4, fraction 5, fraction 6 were combined to part 2; and so on), and then desalted, respectively. Samples were reconstituted in 40 μl 0.1% (v/v) trifluoroacetic acid and stored at −80°C for LC-MS/MS analysis.

### LC-ESI-MS/MS Analysis

Liquid chromatography (LC)-electrospray ionization (ESI) tandem MS (MS/MS) analysis were performed using a Q Exactive mass spectrometer coupled to an Easy nLC (Proxeon Biosystems) according to previous reported literatures with minor modifications (Hu et al., 2015; Zhao et al., 2016). In brief, each fraction was injected for nanoLC-MS/MS analysis. The peptide mixture was loaded onto a reverse phase trap column (Thermo Scientific Acclaim PepMap100) connected to the C18-reversed phase analytical column (Thermo Scientific Easy Column) in 0.1% Formic acid (buffer A) and separated with a linear gradient of 0.1% Formic acid and 84% acetonitrile (buffer B). Using the IntelliFlow technology, the flow rate was controlled at 300 nl/min. The linear gradient was 0–35% buffer B during 0–50 min, 35–100% buffer B for 5 min and hold in 100% buffer B for another 5 min. Positive ion mode was employed in the mass spectrometer. Using data-dependent top 10 method dynamically choosing the most abundant precursor ions from the survey scan (300–1,800 m/z) to acquire MS data. Automatic gain control (AGC) target was set to 3e6 and maximum inject time to 10 ms. Dynamic exclusion duration was 40 s. Survey scans were obtained at a resolution of 70,000 at m/z 200 and the resolution for HCD spectra was set as 17,500 at m/z 200 with 2 m/z isolation width. Normalized collision energy was 30 eV and the underfill ratio was defined as 0.1%. The instrument was run with peptide recognition mode enabled (Hu et al., 2015; Zhao et al., 2016).

### Data Analysis

All the raw data files were converted into MGF format files and searched using MASCOT engine 2.2 (Matrix Science) embedded into Proteome Discoverer 1.4 (Thermo Scientific) against the uniprot_Poaceae database containing 1,440,384 sequences (http://www.uniprot.org, downloaded on March 20, 2017) according to previous description (Hu et al., 2015; Zhao et al., 2016). Mascot search parameters were set as follows: Enzyme: Trypsin; Max missed cleavage: 2; Fixed modification: Carbamidomethyl (C), iTRAQ8plex(N-term), iTRAQ8plex(K); Variable modification: Oxidation (M), iTRAQ8plex (Y); Peptide mass tolerance: ±20 ppm; Fragment mass tolerance: 0.1 Da; Peptide false discovery rate (FDR) ≤1%. Proteome Discoverer 1.4 software was used for data normalization. All peptide ratios were normalized by the median protein. The median protein ratio should be 1.0 after the normalization. The software identified the median average protein ratio and corrected it to unity, and then applied this factor to all quantification results. The quantitative protein ratios were weighted and normalized by the median ratio in Mascot (Tomizioli et al., 2014; Wu et al., 2018). Only those proteins with protein scores over 20 in all biological replicates and having at least two unique peptides were used for further DAP analysis. Protein species with an abundance ratio fold change of at least 1.2 and a *P* < 0.05 were defined as DAPs for further screening (Chu et al., 2015; Chen et al., 2017). The DAPs involved in the processes of LN-promoted PRG and common response to LN stress were defined through a two-step-screening. Only those DAPs that coexist in two specific subsets (DAPs from XY54_LN-XY54_CK comparison and LRG_LN-LRG_CK comparison) or all four subsets were considered to be the final DAPs that truly participate in the corresponding biological process. The identified DAPs were classified based on their biological functions obtained from UniProt database (http://www.ebi.uniprot.org). For the functional uncharacterized DAPs, the putative function of each protein was described according to the annotation of its homologous proteins in other species by using the web-based Basic Local Alignment Search Tool (BLAST, https://www.uniprot.org/).

### Pathway Enrichment Analysis

The pathway enrichment analysis was performed using a web-based tool KOBAS 3.0 (http://kobas.cbi.pku.edu.cn/anno_iden.php) (Xie et al., 2011). The *P*-values were adjusted with Benjamini–Hochberg correction for multiple testing. Only pathways with corrected *P*-values under a threshold of 0.05 were considered as significant.

### Real-Time PCR

Total RNA was extracted using TranZol Up (Transgen, USA). DNase I (Promega, USA) was used to remove genomic DNA. Total RNA extracted from roots was used to generate cDNA samples via random priming with the GoScript Reverse Transcription System (Promega, USA). Six DAPs (including two nitrogen transporters and four randomly selected proteins) were selected for RNA level examination to verify the iTRAQ data and investigate the correlation of protein species abundance with their corresponding mRNA level. The cDNA samples were used as templates and mixed with primers and SYBR Green PCR Real Master Mix (Tiangen, China) for real-time PCR analysis using Thermal Cycler CFX96 Real-Time System (Bio-Rad, USA). The temperature procedure was: 95°C for 15 min followed by 40 cycles of 95°C for 15 s, 60°C for 15 s, and 72°C for 15 s. *TaActin* was used as a reference gene to normalize the expression level of target genes. 2^−ΔΔCT^ method was employed to calculate the relative gene expression level. Three biological replicates were performed. The primers used for real-time PCR were designed using Primer 5.0 software and are listed in Table S1.

### Statistical Analysis

The means, standard deviations (SD), standard errors (SE), and ranges of each measured morphological trait were calculated by using Microsoft Office Excel 2017. IBM SPSS statistics 21 software was used to determine the statistical significance of the data. For each morphological trait and gene expression level, one-way ANOVA analysis and Duncan's multiple range test at *P* = 0.05 was used to identify significant differences. A web-based tool Venny 2.1 was used to generate Venn diagram (http://bioinfogp.cnb.csic.es/tools/venny/index.html).

## Results

### Root Phenotypes Under Different Nitrogen Supply Levels

**Figure 2:**
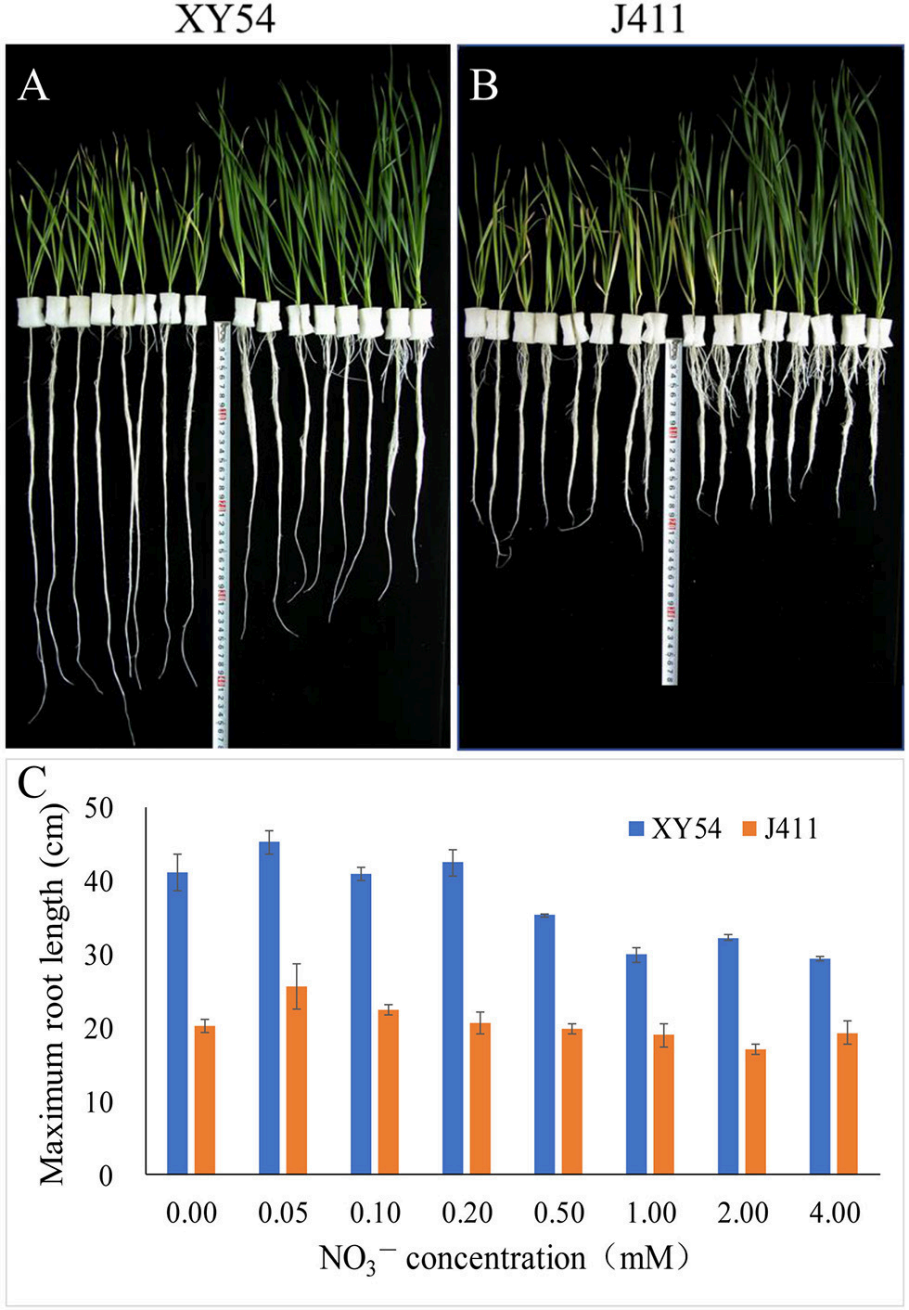
The response of the roots of winter wheat varieties XY54 and J411 to different nitrogen supply levels. The roots and shoots of XY54 **(A)** and J411 **(B)** at 0, 0.05, 0.1, 0.2, 0.5, 1.0, 2.0, and 4.0 mM ${\text{NO}}_{3}^{-}$ concentrations (from left to right, 2 plants per nitrogen supply level) and the maximum root length (MRL) of XY54 and J411 under different nitrogen supply levels **(C)**.

In Trial 1, the female parent XY54 had a significantly longer MRL than the male parent J411 in all treatments (Figure 2). With decreasing nitrogen level, the shoot length decreased gradually in both XY54 and J411, while the roots of XY54 and J411 exhibited a differential response to LN stress (Figures 2A,B). The growth of XY54 primary roots were significantly promoted by 0.2 mM N or even lower N concentration, while this was not obvious in J411. The MRL of XY54 at 0.2 mM N supply level was 31.4 and 44.1% longer than that at 2.0 mM and 4.0 mM N, respectively (Figure 2C). In Trial 2, the female parent XY54 had a significantly longer MRL than the male parent J411 under both CK and LN conditions, which was consistent with Trial 1 and our previous study (Ren et al., 2012). XY54, but not J411, exhibited enhanced PRG under LN conditions. Similarly, LN had obvious induction on the growth of primary roots in the LRG group, but not in the SRG group (Table 1).

**Table 1:** Mean values and ranges for MRL in the LRG group, SRG group, XY54, and J411.

**Trait (cm)**	**Parent (Mean** **±** **SE)**		**LRG group**			**SRG group**		
	**XY54**	**J411**	**Mean ± SD**	**Min**.	**Max**.	**Mean ± SD**	**Min**.	**Max**.
MRL-CK	17.1 ± 0.7(a)	11.4 ± 0.7(b)	12.3 ± 1.9	8.7	17.5	9.8 ± 2.0	7.0	15.2
MRL-LN	20.3 ± 0.8(a)	11.7 ± 0.7(b)	18.4 ± 2.0	15.9	23.6	10.4 ± 2.0	7.5	15.9

*MRL, maximum root length; LRG group, the group of RILs exhibiting longer root growth promoted by low nitrogen was named as LRG group; SRG group, the group of RILs exhibiting shorter root growth promoted by low nitrogen was named as SRG group. Statistical difference is indicated by different letters after the means. Letters (a and b) designate significance at P < 0.05*.

### Protein Identification

The iTRAQ-based quantitative proteome characterization approach was used to investigate the regulatory mechanisms of LN-promoted PRG. Protein profiles of XY54, J411, LRG, and SRG in roots under CK and LN conditions were obtained according to the workflow shown in Figure 1. All the raw mass spectra files in LC-MS/MS have been deposited into the publicly accessible database iProX (id: IPX0001149000/PXD008793. The files named P17093_1, P17093_2, and P17093_3 contain the results of replicate 1, replicate 2, and replicate 3, respectively). In total, 3,075 proteins were identified and found to be present in sufficient amounts to be reliably quantified (FDR ≤ 1%, unique peptide ≥2 and protein score ≥20; Table S2). These proteins were used for the following DAPs analysis.

### Identification of DAPs Involved in the Process of LN-Promoted PRG

**Figure 3:**
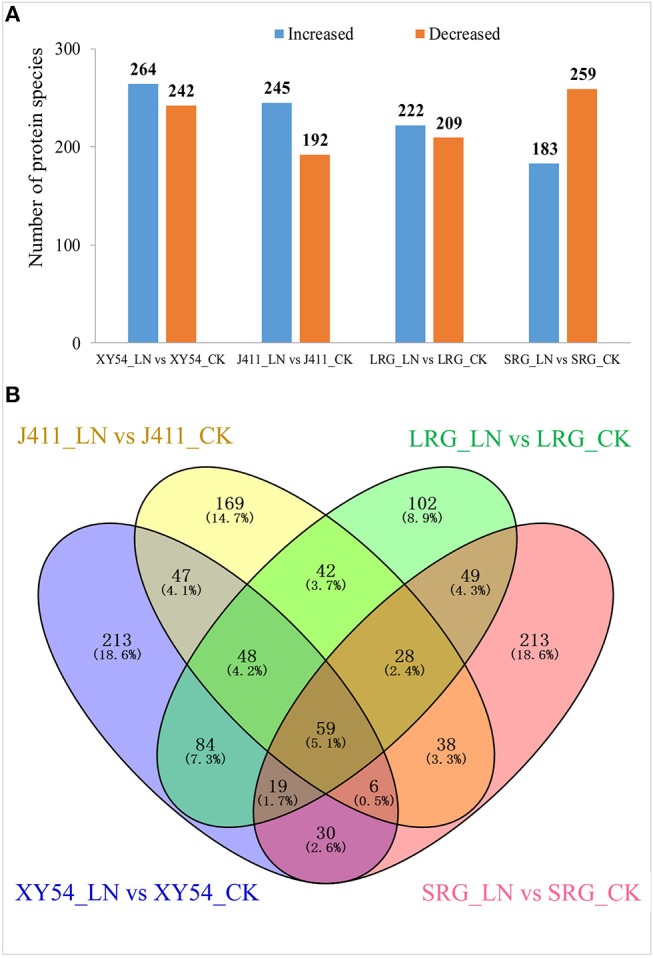
Identification of differentially abundant proteins (DAPs) under CK (control) and low nitrogen (LN) conditions in different genetic backgrounds. **(A)** Number of significantly increased or decreased protein species under LN conditions compared with CK. **(B)** Venn diagram analysis of DAPs in all the one-to-one comparisons between CK and LN.

In one to one comparison between XY54_LN and XY54_CK, 506 proteins showed more than 1.2-fold change (*p* < 0.05) in their respective abundances in roots and were classified as DAPs (Table S3). Among the DAPs, 264 proteins were increased and 242 proteins were decreased in XY54_LN compared with XY54_CK (Figure 3A). In the J411_LN-J411_CK comparison, 437 DAPs were identified. Among them, 245 DAPs exhibited increased abundances and 192 DAPs showed decreased abundances in the roots of J411 under LN conditions compared with CK (Table S4; Figure 3A). In the LRG_LN-LRG_CK comparison, a total of 431 DAPs was identified. Among them, 222 DAPs exhibited increased abundances and 209 DAPs showed decreased abundances in the roots of LRG_LN compared with LRG_CK (Table S5; Figure 3A). In the SRG_LN-SRG_CK comparison, 442 proteins showed more than a 1.2-fold change (*p* < 0.05) in their respective abundance in roots. Among them, 183 DAPs were increased and 259 DAPs were decreased in SRG_LN compared with SRG_CK (Table S6; Figure 3A).

Among the DAPs identified above, 84 DAPs existed in both the LRG_LN-LRG_CK comparison and in the XY54_LN-XY54_CK comparison but were not present in the SRG_LN-SRG_CK comparison and the J411_LN-J411_CK comparison (Figure 3B). In other words, these proteins only exhibited differential accumulation in the roots of XY54 and LRG under LN stress compared with CK (Figure 3B; Table 2). Therefore, there is reason to believe that these 84 DAPs are highly likely to be involved in the process of LN-promoted PRG because most of the differences in the genetic backgrounds of XY54 and J411 had been eliminated.

**Table 2:** List of differentially abundant proteins (DAPs) involved in the process of LN-promoted primary root growth.

**Accession number**	**Description**	**Reference organism**	**Changes**	**Folds changed**		***P*****-value**	
				**XY54_LN/** **XY54_CK**	**LRG_LN/** **LRG_CK**	**XY54_LN/** **XY54_CK**	**LRG_LN/** **LRG_CK**
**STRESS AND DEFENSE**							
A0A1D5ZCY9	Pop3 peptide	*Zea mays*	Up	1.70	1.32	0.035	0.030
A0A1D5YDS7	Germin-like protein 3–7	*Oryza sativa*	Up	1.33	1.23	0.008	0.012
M8B4P2	Heat shock cognate 70 kDa protein	*Aegilops tauschii*	Up	1.24	1.24	0.039	0.037
Q0Q0I7	Heat shock protein 90	*Triticum aestivum*	Up	1.23	1.29	0.032	0.001
A0A1D5XYS1	Heat shock protein 20	*Oryza sativa*	Up	1.21	1.26	0.012	0.029
A0A1D5SNM1	Dirigent protein	*Triticum aestivum*	Down	0.41	0.47	0.012	0.013
A0A1D5UKG2	Wound/stress protein	*Zea mays*	Down	0.67	0.79	0.011	0.015
A0A1D5XQ60	Dirigent protein	*Triticum aestivum*	Down	0.80	0.83	0.021	0.009
**SIGNAL TRANSDUCTION**							
A0A1D6DER2	Elicitor-responsive protein 3	*Triticum urartu*	Up	1.64	1.33	0.009	0.000
P04464	Calmodulin	*Triticum aestivum*	Up	1.54	1.44	0.001	0.024
A0A1D6DDM8	Serine/threonine protein phosphatase 2A	*Triticum urartu*	Down	0.53	0.73	0.031	0.014
A0A1D6ACI4	Receptor-like protein kinase precursor	*Zea mays*	Down	0.67	0.66	0.000	0.001
A0A1D5YA49	Serine/threonine-protein kinase CTR1	*Dichanthelium oligosanthes*	Down	0.73	0.77	0.019	0.042
M8CCZ1	Abscisic acid receptor PYL2	*Dichanthelium oligosanthes*	Down	0.81	0.79	0.007	0.011
**AMINO ACID METABOLISM**							
A0A1D5ZIK7	Aminopeptidase	*Aegilops tauschii*	Up	1.34	1.24	0.003	0.032
A0A1D5U0Y3	Methylmalonate semi-aldehyde dehydrogenase	*Oryza sativa*	Down	0.69	0.80	0.005	0.037
A0A1D5YUR5	Methylcrotonoyl-CoA carboxylase subunit alpha	*Oryza sativa*	Down	0.76	0.79	0.042	0.003
**PROTEIN SYNTHESIS, FOLDING, AND DEGRADATION**							
B0BKB0	Superal1	*Hordeum vulgare*	Up	1.54	1.28	0.002	0.012
A0A1D5TX00	60S ribosomal protein L35-2	*Aegilops tauschii*	Up	1.48	1.23	0.005	0.002
W5CFX4	40S ribosomal protein S24	*Triticum aestivum*	Up	1.47	1.48	0.001	0.027
A0A1D5YZN0	40S ribosomal protein S2	*Oryza sativa*	Up	1.34	1.47	0.043	0.004
W5EPW2	Cathepsin B	*Hordeum vulgare*	Up	1.34	1.36	0.006	0.000
A0A1D5XKL4	26S proteasome non-ATPase regulatory subunit 3	*Zea mays*	Up	1.27	1.21	0.007	0.009
D8L9B3	Protein disulfide isomerase family protein 4-1	*Triticum aestivum*	Up	1.27	1.22	0.001	0.008
W5GI50	60S ribosomal protein L6	*Triticum aestivum*	Up	1.26	1.28	0.003	0.020
A0A1D5TAD1	Anaphase-promoting complex subunit 2	*Aegilops tauschii*	Up	1.23	1.23	0.023	0.017
A0A1D6RZW3	PRA1 family protein	*Triticum aestivum*	Up	1.22	1.37	0.001	0.012
W5DSB4	60S ribosomal protein L28-1	*Aegilops tauschii*	Up	1.20	1.24	0.050	0.003
A0A1D5S955	2-aminoethanethiol dioxygenase	*Triticum urartu*	Down	0.52	0.72	0.018	0.013
A0A1D5TP58	aminoacyl-tRNA synthetase	*Zea mays*	Down	0.70	0.78	0.046	0.002
A0A1D6SEM6	Ubiquitin-like modifier-activating enzyme 5	*Triticum urartu*	Down	0.76	0.75	0.008	0.013
**CARBOHYDRATE AND ENERGY METABOLISM**							
A0A1D6B0P4	Mitochondrial ATP synthase	*Triticum aestivum*	Up	1.49	1.21	0.003	0.043
M7YU16	ATP synthase subunit d, mitochondrial	*Triticum urartu*	Up	1.46	1.31	0.000	0.023
A9U8G1	Alcohol dehydrogenase ADH2D	*Triticum aestivum*	Up	1.37	1.60	0.008	0.005
A0A077RAG2	Cytochrome b5	*Triticum urartu*	Up	1.34	1.24	0.001	0.000
A0A0A9AM13	Atp1, OrsajM_p50	*Arundo donax*	Up	1.33	1.35	0.003	0.004
A0A1D6AKJ1	ADP-ribosylation factor-like protein 8A	*Zea mays*	Up	1.32	1.20	0.002	0.017
Q41629	ADP,ATP carrier protein 1, mitochondrial	*Triticum aestivum*	Up	1.29	1.26	0.005	0.003
A0A1D6B8P9	ADP, ATP carrier protein	*Aegilops tauschii*	Up	1.24	1.33	0.001	0.001
A0A1D5Y4J9	UDP-glucuronic acid decarboxylase 1	*Triticum urartu*	Up	1.22	1.25	0.000	0.003
M7ZDS1	Mitochondrial 2-oxoglutarate/malate carrier	*Oryza sativa*	Up	1.20	1.20	0.000	0.000
F6IB54	Putative feruloyl transferase	*Triticum aestivum*	Down	0.65	0.77	0.033	0.002
A0A1D6RKF2	Aldose 1-epimerase	*Triticum aestivum*	Down	0.72	0.72	0.042	0.021
A0A1D5UN35	AMP-binding enzyme	*Oryza sativa*	Down	0.74	0.77	0.002	0.000
A0A1D5YMM0	Glucose-6-phosphate isomerase	*Triticum aestivum*	Down	0.83	0.82	0.018	0.003
**TRANSPORT**							
A0A1D5SG47	Ras-related protein ARA-3	*Zea mays*	Up	1.45	1.28	0.017	0.002
A0A1D5ZHK0	High affinity cationic amino acid transporter 1	*Aegilops tauschii*	Up	1.29	1.21	0.039	0.020
A0A1D8MIY2	ABCC1	*Triticum polonicum*	Up	1.22	1.24	0.013	0.003
M7YK14	Protein transport protein SEC23-1	*Triticum urartu*	Down	0.75	0.74	0.022	0.028
**REDOX PROCESS**							
A0A1D5ZCL9	Glutathione transferase	*Triticum aestivum*	Up	1.54	1.27	0.006	0.007
A0A1D5YDM0	Peroxidase	*Triticum aestivum*	Up	1.43	1.27	0.015	0.006
A0A1D5UBI2	Thioredoxin	*Triticum urartu*	Up	1.38	1.21	0.012	0.030
G9I6G7	Glutathione peroxidase	*Triticum aestivum*	Up	1.30	1.27	0.003	0.006
M8CKB1	Glutathione transferase F3	*Triticum aestivum*	Up	1.24	1.22	0.016	0.017
W4ZSP8	dihydroflavonol 4-reductase/flavanone protein	*Oryza sativa*	Down	0.48	0.57	0.010	0.014
A0A1D5XHX1	Peroxidase	*Triticum aestivum*	Down	0.64	0.72	0.002	0.003
A0A1D5T090	Minor allergen Alt a 7	*Aegilops tauschii*	Down	0.69	0.80	0.014	0.002
W5AVK6	Peroxidase	*Triticum aestivum*	Down	0.74	0.80	0.008	0.004
A2XFD1	L-ascorbate peroxidase 1	*Oryza sativa*	Down	0.81	0.81	0.030	0.006
A0A1D6AR69	Quinone oxidoreductase PIG3	*Aegilops tauschii*	Down	0.81	0.81	0.035	0.046
**LIPID METABOLISM**							
A0A1D5T8P6	Acyl-[acyl-carrier-protein] desaturase	*Triticum aestivum*	Up	1.20	1.23	0.027	0.003
A0A1D6CPC8	Phospholipase D	*Triticum aestivum*	Down	0.67	0.74	0.015	0.018
A0A1D6CPC7	Phospholipase D	*Triticum aestivum*	Down	0.73	0.80	0.002	0.004
R7WCU6	Phospholipase D	*Aegilops tauschii*	Down	0.75	0.77	0.036	0.002
M0Y397	UCW116, putative lipase	*Hordeum vulgare*	Down	0.76	0.78	0.013	0.014
M8CJ08	GDSL esterase/lipase	*Aegilops tauschii*	Down	0.78	0.76	0.031	0.040
**CHROMATIN STRUCTURE AND RNA PROCESSING**							
A0A1B6PT29	Histone H2A	*Sorghum bicolor*	Up	1.53	1.35	0.001	0.015
I1GV20	Cwf18 pre-mRNA splicing factor	*Brachypodium distachyon*	Up	1.43	1.24	0.003	0.031
A0A1D5WFV2	Histone H2A	*Triticum aestivum*	Up	1.39	1.35	0.017	0.008
M8A374	Histone H4	*Triticum urartu*	Up	1.21	1.23	0.029	0.046
Q8LRU5	HMG-I/Y protein HMGa	*Triticum aestivum*	Down	0.68	0.78	0.004	0.003
R7W1Y6	Protein CLP1 homolog	*Aegilops tauschii*	Down	0.68	0.72	0.028	0.032
A0A1D6A5I5	Enhancer of mRNA-decapping protein 4	*Triticum urartu*	Down	0.72	0.83	0.021	0.038
A0A1D6DBB8	RNA and export factor-binding protein 2	*Aegilops tauschii*	Down	0.75	0.77	0.005	0.015
**OTHERS**							
W5GGG4	O-methytransferase 4	*Triticum aestivum*	Up	1.48	1.21	0.031	0.030
A0A1D5YWZ5	Fasciclin-like protein FLA9	*Triticum aestivum*	Up	1.46	1.4	0.009	0.008
M0XZ30	Reticulon-like protein	*Hordeum vulgare*	Up	1.31	1.30	0.001	0.014
N1QRC7	Adenosylhomocysteinase	*Aegilops tauschii*	Up	1.21	1.24	0.005	0.002
W5GGH2	Expansin EXPB8	*Triticum aestivum*	Down	0.68	0.79	0.005	0.026
A0A1D5VKZ0	Alba DNA/RNA-binding protein	*Zea mays*	Down	0.65	0.79	0.011	0.007
M8CRZ8	BEACH domain-containing protein	*Triticum urartu*	Down	0.70	0.73	0.009	0.013
W5BNV2	TolB protein-related	*Zea mays*	Down	0.71	0.72	0.006	0.001
W5FM07	Thaumatin-like protein TLP5	*Hordeum vulgare*	Down	0.74	0.79	0.003	0.007
A0A1D5X363	DPP6 N-terminal domain-like protein	*Zea mays*	Down	0.76	0.75	0.007	0.002

**Figure 4:**
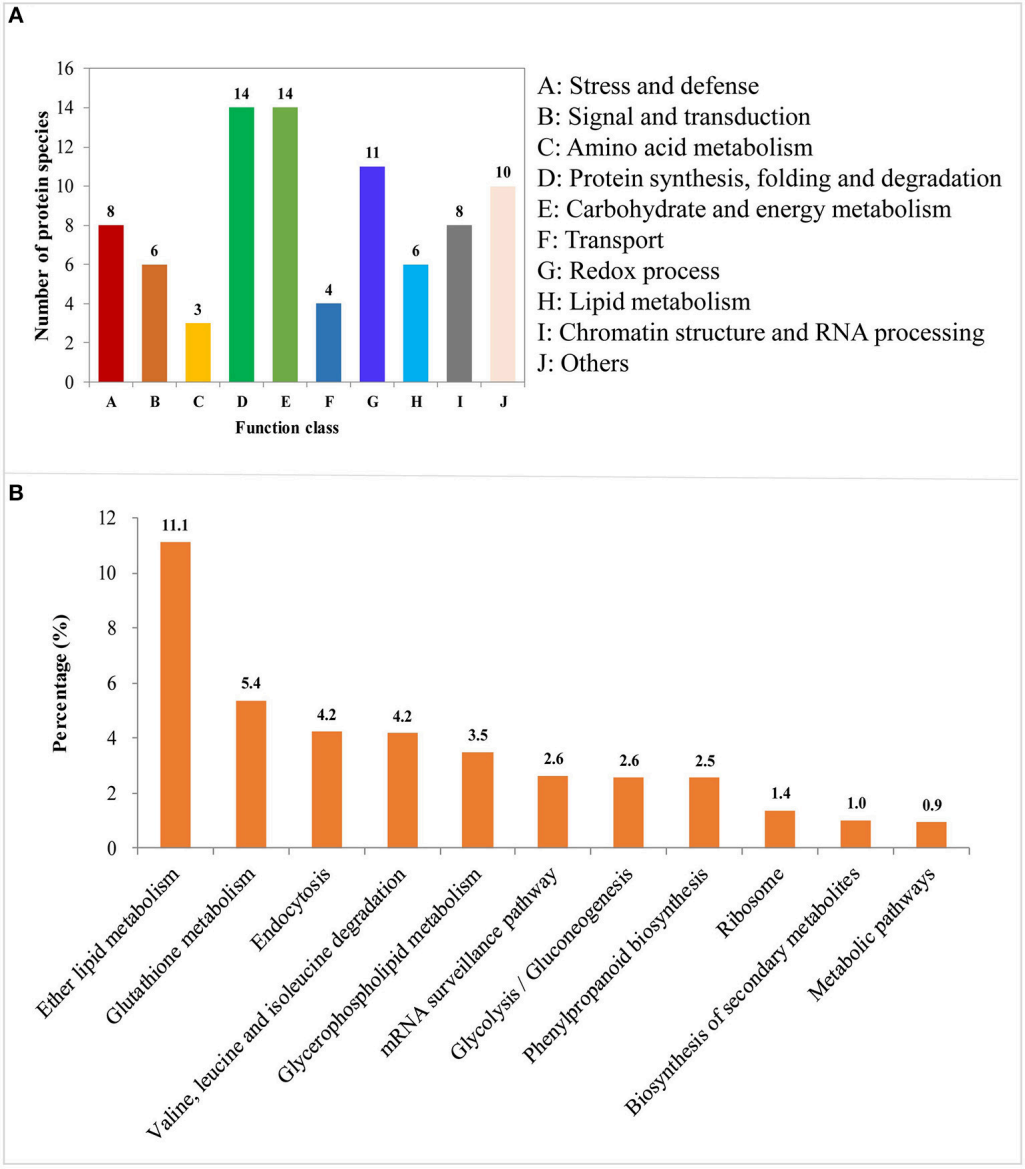
Functional classification and pathway enrichment analysis of the identified differentially abundant proteins (DAPs) involved in the process of LN-promoted primary root growth. **(A)** Classification of the identified DAPs. **(B)** The significantly enriched pathways of the DAPs. The values on the ordinate indicate the percentage of input number of DAPs to the number of the background proteins in the pathway.

DAPs involved in the process of LN-promoted PRG were classified into 10 groups based on their biological functions (Figure 4A; Table 2). The most abundant three groups were “protein synthesis, folding and degradation,” “carbohydrate and energy metabolism,” and “redox process” with 14 DAPs (11.8%), 14 DAPs (11.8%), and 11 DAPs (9.2%), respectively (Figure 4A). The number of DAPs in the groups “stress and defense,” “signal transduction,” “amino acid metabolism,” “transport,” “lipid metabolism,” and “chromatin structure and RNA processing” ranged from three to eight.

### Pathway Analysis of the DAPs Involved in the Process of LN-Promoted PRG

To characterize the metabolic pathways that protein species were implicated in, these DAPs were further investigated using the web-based tool KOBAS 3.0. In total, 11 pathways were significantly enriched in the 84 DAPs (corrected *P* < 0.05) (Table S7; Figure 4B). Among them, most DAPs involved in glutathione metabolism pathway exhibited higher accumulation in XY54 and LRG group under LN conditions, but not in J411 and SRG group. There were five DAPs involved in glutathione metabolism, in which four DAPs were increased (including two glutathione transferases, one aminopeptidase, and one glutathione peroxidase) and one was decreased (L-ascorbate peroxidase 1) in XY54 and LRG group under LN conditions compared with CK. DAPs involved in ether lipid metabolism, beta-Alanine metabolism and mRNA surveillance pathways exhibited higher accumulation in XY54 and LRG group under LN conditions compared with CK. In the other seven significantly enriched pathways, there existed both significantly increased and decreased proteins.

### Identification of DAPs Involved in the Common Response to LN Stress

**Figure 5:**
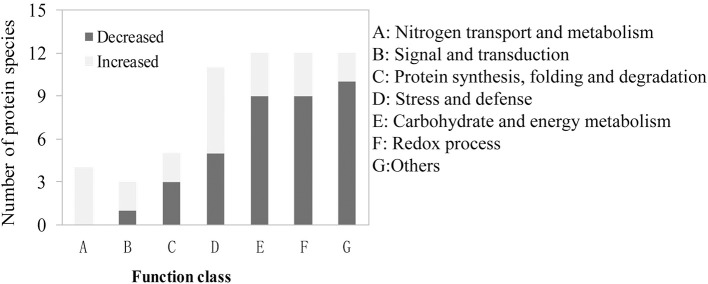
Functional classification of the identified differentially abundant proteins (DAPs) involved in the common response to LN stress.

We also found that 59 DAPs existed in all the four groups of the one to one comparison mentioned above and all of them exhibited similar expression patterns among the four groups (Table S8). These 59 DAPs are likely to be involved in the common response to LN stress in different genetic backgrounds. We classified them into seven groups based on their biological functions (Figure 5). The top three abundant groups were “redox process,” “carbohydrate and energy metabolism” and “stress and defense” with 12 DAPs (20.3%), 12 DAPs (20.3%), and 11 DAPs (18.6%), respectively (Figure 5; Table S8). There also existed four DAPs involved in the process of nitrogen transport and metabolism, and all of them were up-regulated under LN conditions. The proportions of DAPs involved in “signaling and transduction” and “protein synthesis, folding and degradation” were relatively lower than other groups (Figure 5; Table S8). Besides, there also existed 12 DAPs with their functions unclassified.

### Real Time PCR Verification

**Figure 6:**
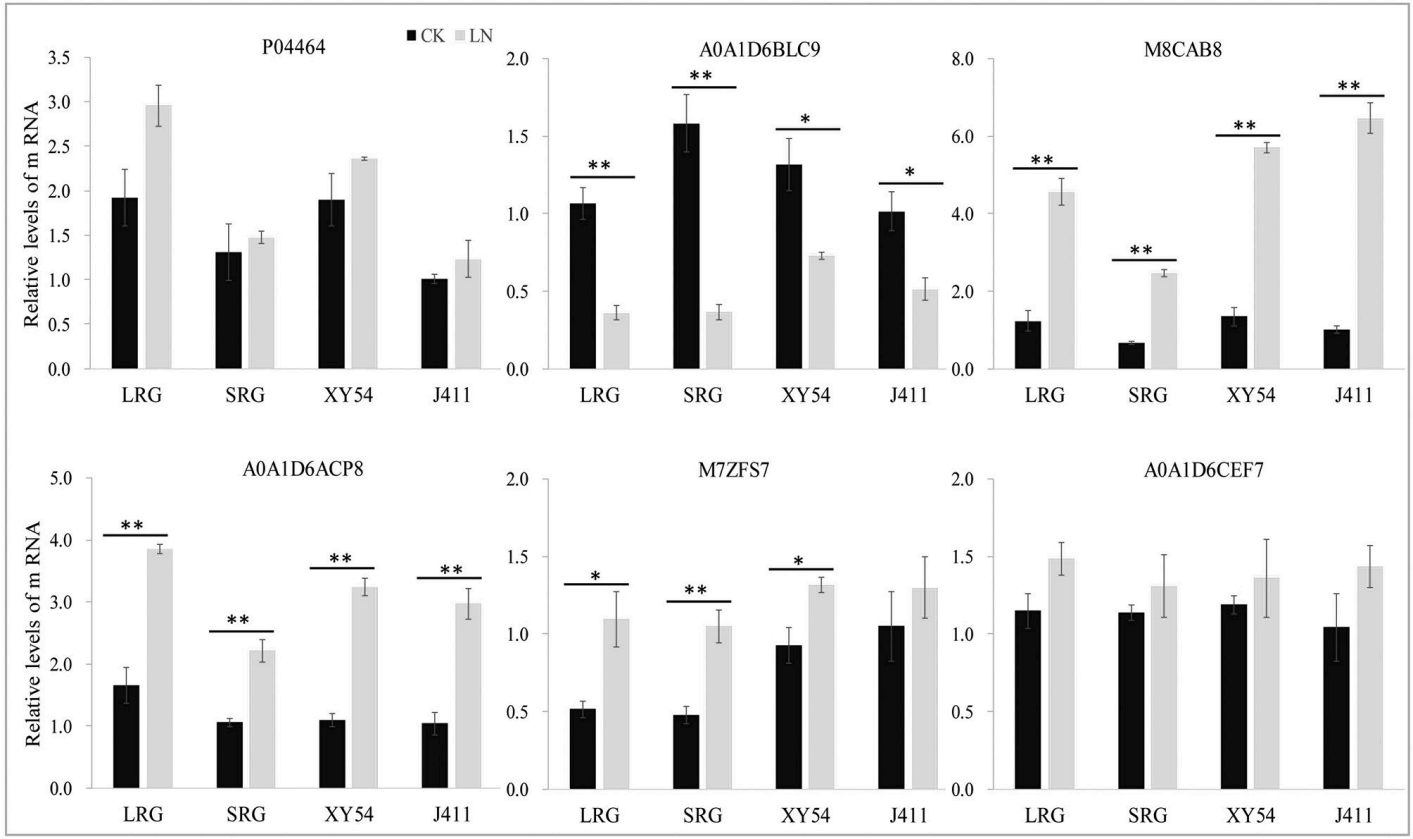
Relative mRNA expression analysis using RT-qPCR on six protein species under control (CK) and low nitrogen (LN) conditions. The expression level in the roots of wheat variety J411 was set to a value of 1. Each bar shows the mean ± standard errors (SE) of three biological replicates. The relative expression level of each gene was calculated using the formula 2 ^−ΔΔCt^ (^*^p < 0.05, ^**^p < 0.01, Duncan's multiple range test).

Of the six selected DAPs, four genes matched well for mRNA level with their translation products, while the other two genes (P04464 and A0A1D6CEF7) exhibited poor correlations between mRNA and protein expression levels (Figure 6). Therefore, post-transcriptional, translational and post-translational regulation may occur in the processes of expression of those poorly correlated genes.

## Discussion

Plants can adapt in response to different types of nutrient deficiency. Nitrogen (mainly in the form of nitrate and ammonia) is highly mobile in soils and readily leached. Low nitrogen availability limits plant shoot growth, while it promotes plant root growth, enabling the root system to reach deeper layers of the soil (Linkohr et al., 2002; Gruber et al., 2013; Lynch, 2013). Therefore, understanding the molecular mechanisms controlling the response of roots to LN stress, especially in food crops, is important for the genetic improvement of root traits and nitrogen use efficiency.

In wheat, the PRG is promoted by LN availability, but there are significant genotypic effects (Ren et al., 2017). For example, LN treatment increased the primary root length and the MRL of wheat variety XY54 but did not significantly influence those of J411 (Ren et al., 2017; Figure 2). Although the phenotype of wheat roots responding to LN has been well-studied, the molecular mechanisms of LN-promoted PRG in wheat remains largely unclear. Here, an integrated comparative root proteome study of the SRG and LRG groups, together with their parents (XY54 and J411) was conducted under CK and LN conditions to extend our knowledge of the regulatory mechanisms. As mentioned above, the DAPs involved in the processes of LN-promoted PRG and common response to LN stress were defined through a two-step-screening. This is done to reduce the interference caused by differences in genetic background of XY54 and J411. In the first round of screening, a relatively lower cut-off, 1.2-fold change (*P* < 0.05), was used to define DAPs. If higher cut-offs were used here, some well-known LN responsive proteins (N1R4I5, A0A1D5UUZ4, etc.) would be missed. As the relative abundance of each DAP in one group of comparison can be verified in another in the second round of screening, a 1.2-fold change cut-off was adopted here to expand the scope of DAP candidates in the first round of screening. Based on this criterion, we ultimately obtained 84 DAPs involved in the process of LN-promoted PRG and 59 DAPs involved in the common response to LN stress were identified (Figure 4A; Table 2). To verify the iTRAQ data and investigate the correlation of protein species abundance with their corresponding mRNA level, six proteins were selected for RNA level examination. Four of them matched well, while the other two genes (P04464 and A0A1D6CEF7) exhibited poor correlations between mRNA and protein expression levels (Figure 6). In addition to the different sensitivities between RT-qPCR and iTRAQ technology, post-transcriptional, translational and post-translational regulation may occur during the expression of those poorly correlated genes. This is consistent with previous studies (Fernie and Stitt, 2012; Chu et al., 2015; Sun X. C. et al., 2017).

Totally, 11 pathways were significantly enriched in the 84 DAPs for the regulation of LN-promoted PRG (Figure 4B; Table S7). For the glutathione metabolism pathway, four DAPs were up-regulated (two glutathione transferases, one aminopeptidase, and one glutathione peroxidase) and one was down-regulated (L-ascorbate peroxidase 1) (Figure 4B; Table S7). Previous studies showed that reduced glutathione (GSH) is required for the maintenance of root apical meristem (RAM) activity and root development (De Tullio et al., 2010; Yu et al., 2013). *Arabidopsis ROOT MERISTEMLESS1* (*RML1*) encodes the first enzyme of GSH biosynthesis, gamma-glutamylcysteine synthetase, which has been shown to be important for regulating cellular redox states. The mutants of *RML1* are unable to establish active root meristems (Vernoux et al., 2000). Moreover, the lateral root (LR) density is also regulated by glutathione redox status. In GSH synthesis mutants, such as *cad2-1, pad2-1, rax1-1*, LR density significantly decreased. Exogenous application of the SL analog increased root glutathione content in wild-type seedlings but not in the SL signaling mutant, *max2-1*, indicating that the role of glutathione in regulating root architecture is linked to SLs (Marquez-Garcia et al., 2014). The glutathione-ascorbate cycle is known as a hydrogen peroxide reducing system (Noctor and Foyer, 1998), in which glutathione acts as a redox buffer against reactive oxygen species (ROS), helping to maintain a reducing environment *in vivo*. This is supported by the finding that differences in accumulation of ROS in the root tip significantly affect root growth (Dunand et al., 2007; Tsukagoshi et al., 2010; He et al., 2014).

Among the 84 DAPs, three proteins were annotated as phospholipase Ds (PLDs), and all of them were involved in multiple enriched pathways (the ether lipid metabolism, glycerophospholipid metabolism, endocytosis, secondary metabolites biosynthesis and metabolic pathways) (Figure 4B; Table S7). Therefore, it was not sure which pathway(s) were involved in the regulatory network of LN-promoted PRG. However, previous studies have shown that PLDs do participate in the regulation of root elongation under varying conditions (Li et al., 2006; Lanteri et al., 2008; Hong et al., 2009). *Arabidopsis* PLDs, PLDζ1, and PLDζ2, were involved in root elongation under low phosphorus conditions. The elongation of primary roots in PLDζ1 and PLDζ2 double knockout mutants was slower than that of wild type and single knockout mutants (Li et al., 2006). Moreover, overexpression another *PLD* gene, *PLD*ε, promoted PRG under severe nitrogen deprivation. The results suggest that PLDs promote organism growth and play a role in nitrogen signaling (Hong et al., 2009). There are four major classes of phospholipases, termed PLAs, PLBs, PLCs, and PLDs, which are distinguished by the type of reaction which they catalyze. PLDs cleave phospholipids after the phosphate, releasing phosphatidic acid. Both phosphatidic acid and its derivative (DAG) are known to be signaling lipids (Hodgkin et al., 1998; Wakelam, 1998). Therefore, the lipid metabolism process may play a key role in connecting membrane sensing of nitrogen status to plant root growth.

The endocytosis pathway with six DAPs was also significantly enriched (Figure 4B; Table S7). Endocytosis is a cellular process in which substances are carried into cells. Cells can sense their environment through endocytosis, gain nutrients, and fine-tune their growth status (Tooze et al., 2014). As a matter of fact, the endocytic pathway intersects with the autophagic pathway when autophagosomes fuse with multivesicular body, forming an amphisome (Eskelinen et al., 2011; Tooze et al., 2014). Studies have shown that autophagy is essential for nutrient recycling and remobilization, and is induced by nitrogen starvation (Guiboileau et al., 2013; Masclaux-Daubresse, 2016). The *Arabidopsis* autophagy-defective (*atg*) mutants exhibit major disorders in nitrogen, carbon, and redox metabolisms, and over-accumulation of specific ribosomal protein subunits (Guiboileau et al., 2013). The altered C and N status in autophagy mutants might result from the rerouting of carbon resources in the phenylpropanoid pathway and amplify oxidative stress in autophagy mutants (Masclaux-Daubresse, 2016). Moreover, overexpression of an *ATG* gene, *SiATG8a*, in foxtail millet under nitrogen starvation conditions resulted in larger roots and higher total nitrogen accumulation than wild-type plants (Li et al., 2015). Interestingly, the phenylpropanoid and ribosome pathways were also significantly enriched for the LN-promoted root growth process in our study (Figure 4B). This is consistent with the previous studies in autophagy mutants (Guiboileau et al., 2013; Masclaux-Daubresse, 2016). These results suggest that endocytosis and autophagy are likely involved in the regulation of LN-promoted root growth. The enrichment of ribosomal proteins means that the rate of protein synthesis and degradation under nitrogen starvation conditions has changed, and this change may be primarily for the recycling of nitrogen in plants. Phenylpropanoids contribute to many aspects of plant responses toward biotic and abiotic stimuli and are very important for plant fitness (Vogt, 2010). Exogenously applied cinnamic acid can influence the phenylpropanoid pathway, resulting in an increase in lignin. These metabolic responses lead to the stiffening of the cell wall and are followed by a reduction in root growth (Salvador et al., 2013). Moreover, phenylpropanoids have been shown to be involved in drought-induced wheat root growth (Dalal et al., 2018). Therefore, the significant enrichment of the phenylpropanoid biosynthesis pathway in the DAPs suggests that similar regulatory mechanism also exist in LN-promoted root growth.

Besides, we also identified 59 DAPs involved in the common response to LN stress (Table S8). The DAPs involved in nitrogen uptake and transportation (NRT2.5, NAR2.1 and an unnamed ammonia transporter) were all significantly increased under LN stress (Table S8). NRT2.5 is a high-affinity nitrate transporter, while NAR2 acts as a partner protein of NRT2 proteins, which is essential for high-affinity nitrate transport system (Tong et al., 2005; Araki and Hasegawa, 2006; Feng et al., 2011; Yan et al., 2011; Kotur and Glass, 2015). In our study, NRT2.5 and NAR2.1 in all the four materials have more accumulation under LN conditions (Figure 5; Table S8), indicating that similar mechanism also exist in wheat. We also observed the DAPs involved in “protein synthesis and degradation,” “carbohydrate and energy metabolism,” and stress response process (Figures 4, 5). These DAPs most probably involved in the regulation of C/N balance and nitrogen remobilization under LN conditions. Therefore, the DAPs involved in the common response to LN stress in wheat roots could enhance the capability nitrogen uptake, transportation and remobilization.

## Conclusion

In this study, we identified 84 DAPs specifically involved in the process of LN-promoted PRG. Pathway enrichment analysis showed that LN-promoted PRG is regulated by a complex set of pathways. The glutathione metabolism, endocytosis, lipid metabolism, and phenylpropanoid biosynthesis pathways may play crucial roles in the regulation of LN-promoted PRG. Some of the DAPs in these pathways (such as the PLDs, glutathione transferases) may be of high value in genetic improvement of wheat root traits and nitrogen use efficiency. However, what are the exact functions of these DAPs in regulating LN-promoted PRG? How nitrogen starvation signals integrate with these pathways to regulate root growth? Further research is needed to generate transgenic lines that overexpress and/or underexpress these DAP-encoding genes to better understand the regulatory mechanisms of LN-promoted PRG.

## Data Availability

All data related to this study has been deposited into the publicly accessible database iProX (www.iprox.org) with identifier IPX0001149000/PXD008793.

## Author Contributions

YX and YR performed most of the experiments. JL, LL, and SC took part in partial work of this research. ZW, ZX, FC, and YT gave many advices during the research. YR, TL, and DC designed the experiments. YR and YX wrote the paper.

### Conflict of Interest Statement

The authors declare that the research was conducted in the absence of any commercial or financial relationships that could be construed as a potential conflict of interest.
